# Application of Foot Hallux Contact Force Signal for Assistive Hand Fine Control

**DOI:** 10.3390/s23115277

**Published:** 2023-06-02

**Authors:** Jianwei Cui, Bingyan Yan, Han Du, Yucheng Shang, Liyan Tong

**Affiliations:** School of Instrument Science and Engineering, Southeast University, Nanjing 210096, China; 220213676@seu.edu.cn (B.Y.);

**Keywords:** continuous fine actions’ control, posture control, contact force signals, assistive hand, upper limb assistive robots, human–robot interaction, wearable sensors

## Abstract

Accurate recognition of disabled persons’ behavioral intentions is the key to reconstructing hand function. Their intentions can be understood to some extent by electromyography (EMG), electroencephalogram (EEG), and arm movements, but they are not reliable enough to be generally accepted. In this paper, characteristics of foot contact force signals are investigated, and a method of expressing grasping intentions based on hallux (big toe) touch sense is proposed. First, force signals acquisition methods and devices are investigated and designed. By analyzing characteristics of signals in different areas of the foot, the hallux is selected. The peak number and other characteristic parameters are used to characterize signals, which can significantly express grasping intentions. Second, considering complex and fine tasks of the assistive hand, a posture control method is proposed. Based on this, many human-in-the-loop experiments are conducted using human–computer interaction methods. The results showed that people with hand disabilities could accurately express their grasping intentions through their toes, and could accurately grasp objects of different sizes, shapes, and hardness using their feet. The accuracy of the action completion for single-handed and double-handed disabled individuals was 99% and 98%, respectively. This proves that the method of using toe tactile sensation for assisting disabled individuals in hand control can help them complete daily fine motor activities. The method is easily acceptable in terms of reliability, unobtrusiveness, and aesthetics.

## 1. Introduction

According to the data of the Second National Survey on Disabled People of the People’s Republic of China in 2007, there are 24.12 million people with physical disabilities in China [1]. Upper limb disabilities bring many inconveniences to people’s daily lives, and for those with severe disabilities, they may even be unable to live independently, which seriously affects their mental health. People with hand disabilities often desire a prosthetic hand that has the same functionality as a human hand, and related research in the field of intelligent control is also an important research topic [2,3,4]. The intention recognition of the human brain and the rich sensory perception of human-like nerves are the basis for the functionality of prosthetic hands. In addition, the comfort and aesthetic appearance of prosthetic hands are also important factors that affect their promotion and application.

At present, the signal sources that can express the human brain’s intention to control the disabled hand are mainly electromyography (EMG) signal, electroencephalogram (EEG) signal, voice signal, and motion signal. All aforementioned control methods attempt to infer human upper limb movement intention through means such as neural networks, rendering them less precise and vulnerable to interference. In this paper, we propose an active approach for acquiring upper limb movement intention, whereby the user simply presses the smart insole according to a pre-agreed mapping relationship table to execute various assistive hand movements. 

The first problem solved in this paper is how to obtain a stable control signal in accordance with the intention of the human brain from the fluctuating plantar contact force signal, and the other problem is how to control the working posture of the assistive hand. When the assistive hand manipulates small and soft objects (such as tying shoelaces), the posture of the fingers should be accurate. If the posture error is large, the tremor of the human arm and the assistive hand and the uncoordinated displacement of the assistive fingers cause the assistive hand to fail to grasp the target object. When non-disabled people grasp objects, they will naturally control the relevant position and distance between the hand and the target to achieve very fine operation, which is actually a natural force-position control process. There are also many studies on the soft grasping of the assistive hand [5,6]. Unfortunately, the structure of the assistive hand is complex, limited in size, and also limited by cost requirements. There are no force-position measurement and control components built into the commercial assistive hand. Another issue investigated in this paper is how to achieve anthropomorphic fine manipulation based on the existing assistive hand.

This paper mainly solves the problems existing in the assistive hand, which are as follows: (a) In view of the characteristics of poor stability and susceptibility to interference in the methods of controlling the assistive hand such as EMG signal and EEG signal, this paper uses the foot contact force signal as the control signal, and the disabled person only needs to press the insole to control the manipulator, which has good stability and anti-interference, as verified by experiments. (b) In investigating the needs of disabled people for disability equipment, it was found that they have high requirements for ease of use and unobtrusiveness. This method places intelligent insoles in shoes, and different modules communicate through wireless means, which can better meet their needs.

In order to solve the above problems, this paper proposes an interactive control method for the assistive hand based on foot contacts, which has achieved very effective results. The main work of this paper is as follows.

(1) Propose and design a method to obtain human control intentions. The foot haptic signal is selected as the control signal, and the hallux (big toe) haptic signal is selected by analyzing the characteristics of signals in different areas of the foot. By using characteristic parameters such as peak number to characterize signals, stable control signals corresponding to human intentions can be obtained from fluctuating contact force signals, which can significantly express human grasping intentions.
(2) Develop a fine action control method for the assistive hand. The mathematical model of the assistive hand has been established to control the gesture of fingers of the assistive hand without the use of position measurement and control equipment, and accurately grasp objects of different sizes, different shapes, and different hardness.
(3) Combine the human brain’s intention recognition with the fine control of the assistive hand. Many human-in-the-loop experiments are conducted using human–computer interaction methods, which shows that assistive hands efficiently perform a variety of daily activities and help people with hand disabilities to live normal lives.

The rest of the paper is organized as follows. In Section 2, we present an overview of the current research on assistive hand control methods and their background. In Section 3, experiment devices are introduced, and the appropriate area of the foot is screened to generate the contact force signal as the control signal. Section 4 introduces the processing and extraction method of force signals. Section 5 designs a fine action control method for the assistive hand. In Section 6, people with hand disabilities are invited to perform daily activities by using the above methods. Section 7 shows the experimental results and Section 8 concludes the paper.

## 2. Background

### 2.1. Summary of Research Background

There are many studies on assistive hand control and human intent recognition, and we summarize the major ones in Table 1 and show the data sources and applications.

### 2.2. Common Methods for Recognizing Upper Limb Movement Intentions

The key to assistive hand control lies in the recognition of human upper limb movement intentions. Currently, common methods for recognizing intention utilize biological signals such as EMG and EEG signals.

The principle of EMG signal control is that when human upper arm muscles contract, they produce obvious electrical signals that have a strong coupling relationship with hand movements and can be used to control the assistive hand. Feleke et al. [2] used EMG signals to predict the three-dimensional position of the hand and analyze complex trajectories of the hand. Zheng et al. [3] used EMG signals to identify and classify hand movements and used a sliding window of long-term memory to investigate continuous hand motion prediction methods. Nazari et al. [4] reviewed the use of EMG signals to control the assistive hand, and they used ultrasonic sensing to control a bionic hand and machine learning to classify hand gestures. Jiang et al. [7] used EMG signals and IMU to recognize gestures and combined various models such as LSTM to improve the recognition accuracy. Zhang et al. [8] used a machine learning approach to process surface EMG signals at the forearm and hand muscles for the recognition of six gestures. Yang et al. [9,23] proposed a scalable two-stage machine learning gesture recognition framework and used multivariate variational modal decomposition to extract poor features on EMG signals, which can recognize 52 gestures. Triwiyanto et al. [10] developed a real-time embedded time-domain feature extraction and machine learning algorithm with good accuracy for assistive hand control. EMG signals contain a wealth of information regarding the intention of upper limb movements, and are effectively utilized in identifying hand movements and gesture classification. However, the EMG signal is susceptible to interference from factors such as electrode placement, muscle atrophy, perspiration, and other variables. As a result, it may not accurately reflect the intended movements of individuals with hand disabilities and can lead to erroneous actions by assistive hands. Additionally, EMG signal acquisition devices are tightly affixed to the skin of the human body, which can cause discomfort. Furthermore, acquiring EMG signals necessitates muscle tension and may prove arduous for users to regulate, resulting in low acceptance rates. 

The EEG signal is also a very popular topic in the field of disability. When the human brain imagines some limb movements, a weak electric field is generated in the brain. According to the distribution of the electric field, the assistive hand can reproduce some movements. Wang et al. [11] investigated the neural features of the main hand motion directions, obtained by decoding EEG signals under different hand motion conditions, and developed a new decoding method based on nonlinear kinetic parameters of motion-related cortical potentials (MRCPs). Fu et al. [12] designed multiple patient-appropriate motor tasks in an EEG-based trial of BCIs, incorporating robotic, functional electrical stimulation (FES), or other combined feedback to help patients perform grip, finger extension, thumb-to-thumb, or other movements. Ofner et al. [13] determined hand motion information by extracting time-domain features of EEG signals, including postures such as opening and grasping. However, the extraction of EEG signal characteristics poses a challenge and requires users to focus on imagining limb movements in an interference-free environment, thus hindering current applications.

### 2.3. Application of Smart Insoles

Feet are flexible organs. Many people with hand impairments can use their feet to perform activities such as writing and eating. Is it possible to control the assistive hand with feet [24] In recent years, smart insoles have been widely used in foot posture detection and sports health analysis. Tang et al. [14,15] used smart insoles for human health analysis and extracted the corresponding features by analyzing plantar pressure data. However, there is a lack of research on smart insoles as a signal source to control the assistive hand. The smart insole has the characteristics of low power consumption, ease of use, and strong wearing ability, and it does not affect people’s appearance, which meets the aesthetic requirements of assistive hands. Based on the above consideration, this paper tries to use a smart insole to control the assistive hand.

### 2.4. Other Control Methods for Assistive Hands

Voice signals can also control the assistive hand to complete the movement. Vijayaragavan et al. [16] designed, analyzed, and fabricated actively controlled prosthetic hands suitable for complex gestures and developed a mobile application with Bluetooth connectivity for voice control. However, it is susceptible to environmental noise interference.

Disabled people hope that the assistive equipment cannot be detected by others and can be worn comfortably [25]. It is found that people’s daily activities have fixed operation objects, locations, and methods. For example, the action of putting on a coat is in the upper part of the human body, and the action of drinking water is to grab an object somewhere outside the body and send it to the mouth. Therefore, using acceleration sensors to measure the movement of the human arm and combining it with machine vision information can also reflect the manipulation intention of the assistive hand to a certain extent, and can be used to control the movement of the assistive hand. These sensors can be hidden in clothing in the form of wearable devices and do not need to be in close contact with the human skin, which has advantages in terms of comfort and aesthetics. Cui et al. [17,18] used IMU to identify upper limb motion intention and extract upper limb pose information features combined with long short term memory (LSTM) to predict motion trajectories. Li et al. [19] used EMG and motion signals combined with convolutional neural networks (CNN) to achieve the classification of human movement patterns. Currently, the stability of this type of technology is not very high. Chakraborty et al. [20] used image segmentation and face feature detection to study motion and line-of-sight estimation and proposed a new machine learning method to improve the accuracy of recognition. Qin et al. [26] designed a magnetic array-assisted sliding friction electrical sensor that detected hand flexion and extension movements as well as the direction and speed of motion. Rasel et al. [21] used image information for gesture recognition, combined with machine learning methods for gesture classification. Sarac et al. [27] detailed each option by surveying the design choices available for exoskeletons and highlighting their impact on the type of application. Sarcar et al. [22] used visual sensors to obtain information about human arm movements and used a combination of CNN and LSTM to classify arm movements into violent and nonviolent categories. These methods have good results in terms of accuracy, but their wearability does not meet the requirements of people with disabilities.

## 3. System Design

### 3.1. Hardware Device for the Assistive Hand

**Figure 1:**
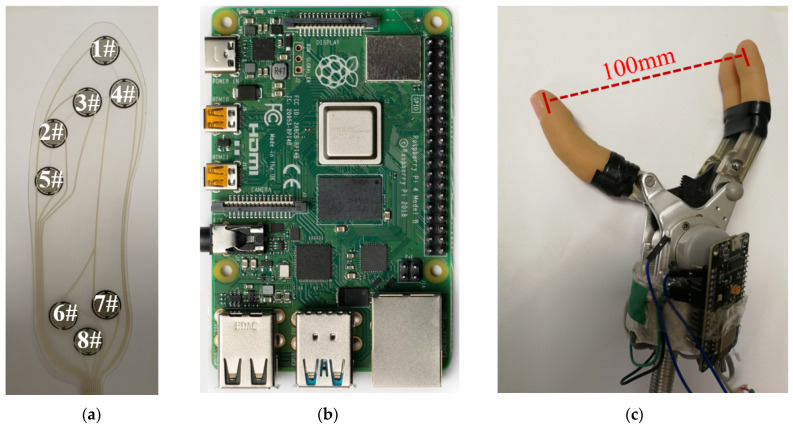
Experimental equipment. 1# to 8# are the sensor numbers of the insole. (**a**) Smart insole; (**b**) computer; and (**c**) manipulator.

Based on the above ideas, this paper carries out research on the foot contact control method. The human foot is mainly composed of the hallux, the little toe, the ball of the foot, the arch of the foot, and the heel [28]. Studies have shown that the normal arch of the foot is in a concave state compared to the insole plane, and the interaction force with the insole is very small. The heel and sole of the foot can generate force signals only when the sole of the shoe touches the ground or other objects, and the signals from these two parts can characterize the person’s walking state. The hallux and the little toe can generate force signals without the help of other objects, which is highly flexible. Force-sensing elements are arranged in the insole area corresponding to the hallux, little toe, ball of the foot, and heel to detect the contact force of the corresponding parts. As shown by the black dot number in Figure 1a, the flexible smart insole selected in this paper has flexible contact force sensors distributed in the hallux (1#), little toe (2#), forefoot (3#, 4#), and heel (6#, 7#, 8#). The model of the smart insole in Figure 1a is ZNX-01, a flexible thin-film pressure sensor produced by Suzhou Leanstar Electronic Technology Co., Ltd., Suzhou, China [29]. The smart insole and the AD conversion module are purchased by us, and what we do is connect them. Figure 1b shows the Raspberry Pi 4B, running on the Ubuntu 18.04 LTS operating system. The model of the artificial hand in Figure 1c is SJQ12-F [30], produced by Danyang Prosthetic Limb Factory Co., Ltd., Danyang, China.

The artificial hand selected for this system is very sensitive and easy to control; it uses high and low level signals for control, corresponding to open/close and stationary respectively, so interfinger distance of the artificial hand can be controlled by a continuous input signal. The control logic of the artificial hand is shown in Table 2, and the following conclusions can be drawn: (a) when a high level signal is input to line A and a low level signal is input to line B at the same time, the artificial hand will gradually open; (b) when a low level signal is input to line A and a high level signal is input to line B at the same time, the artificial hand will gradually close; (c) when low level signals are input to both line A and line B, the artificial hand remains stationary. Combining (1), (2), and (3), i.e., controlling the duration of the corresponding input signal, can control the artificial hand at a certain interfinger distance.

In order to study the foot contact force information, the test equipment selected in this paper is shown in Figure 1. The smart insole is shown in Figure 1a, which can measure the contact force distribution. Figure 1b shows the force signal processing and communication module. Figure 1c shows the core module of the three-finger single-degree-of-freedom manipulator produced by China Danyang Prosthesis Factory. When they use the assistive hand, plastic rings and little fingers can be installed to make it more similar to the appearance of a human hand. By finding information about manipulators on the market, due to cost, volume, and other reasons, manipulators do not have independent position measurement and control components [31,32], and the full opening size is 100 mm. The single-degree-of-freedom opening and closing movements rely only on limiting the current of the drive motor to realize the clamping function of the thumb and index finger [31,32]. We designed a communication scheme between the smart insole, manipulator, and Raspberry Pi, and wrote programs to implement the data transfer function between them. The smart insole and the computer transmit data through wireless communication, and the computer and the manipulator also transmit data through wireless communication. The computer sends control signals to the manipulator. 

### 3.2. Introduction of Research Methods

**Figure 2:**
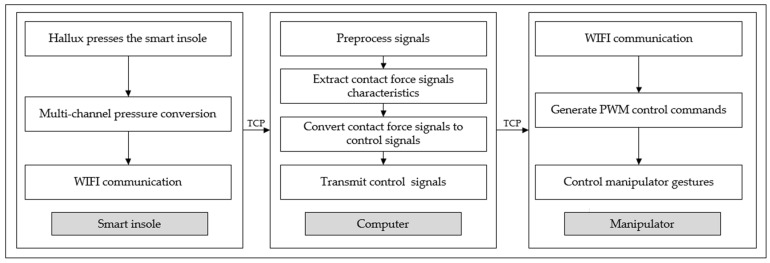
Flow chart of the model.

We propose an assistive hand control method with the flow chart shown in Figure 2, mainly using devices shown in Figure 1. Firstly, the user’s hallux presses the smart insole, the multi-channel pressure conversion module is responsible for collecting and converting signals, and the signal is transmitted to the computer by the WIFI module through TCP communication. Then, the computer is responsible for processing the signal, which mainly includes the following steps: (a) preprocess and filter the signal; (b) the contact force signal features are extracted to obtain the pressing times; (c) the contact force signal characteristics are transformed into the control signal, namely the interfinger distance, which is mapped to the grasping posture; (d) the control signal is transmitted to the manipulator through TCP communication; and, finally, the manipulator is controlled to complete the action, and the PWM signal control method is used to convert the control signal into the opening and closing instructions of the manipulator.

### 3.3. Information on Tests

In both Section 3.4 and Section 4.3, we invited volunteers to participate in the test to verify the validity of methods. Volunteers were divided into non-disabled individuals and those with hand disabilities only, with non-disabled individuals recruited from colleagues and those with hand disabilities recruited with the help of a disability federation. The volunteers were asked to place the smart insoles into the shoes and then press different areas, as detailed in Section 3.4 and Section 4.3. Once the smart insoles are pressed, contact force signals are generated and then mapped for data analysis. We invited 10 volunteers. Their information was shown in Table 3. All subjects gave their informed consent for inclusion before they participated in the study. The study was conducted in accordance with the Declaration of Helsinki, and the protocol was approved by the Ethics Committee of XDJK2020B029, SWU41015718, SWU20710953.

### 3.4. Investigation of the Foot Contact Force Signal

**Figure 3:**
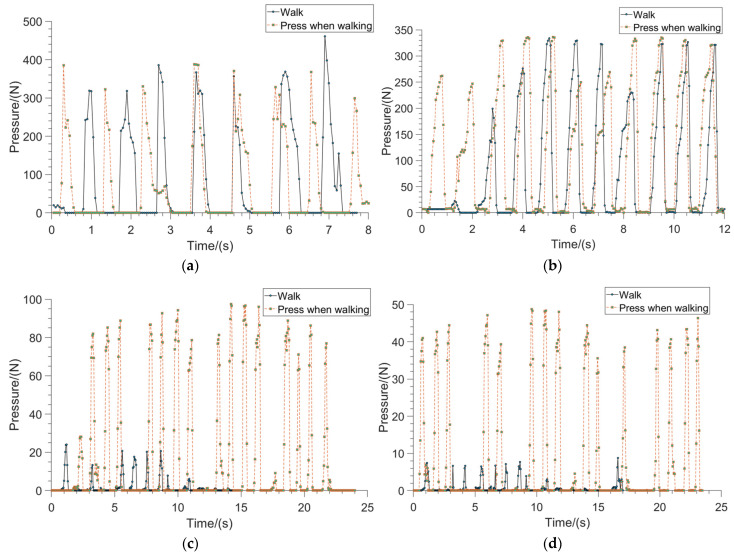
Four area contact force curves of smart insole during walking. (**a**) Contact force curve of heel area; (**b**) contact force curve of forefoot area; (**c**) contact force curve of hallux area; (**d**) contact force curve of the little toe area.

There are two main states of lower limbs in common activities: rest and movement. Walking and running are the main states of motion. The smart insole is used to collect the contact force signals from four parts of the foot: the heel, forefoot, hallux, and little toe during the walking state. Ten volunteers were invited to independently complete walking as well as pressing the four regions while walking respectively. It was found that these 10 sets of contact force data obtained showed similar characteristics, so one set of data was selected to analyze its characteristics, as shown in Figure 3. The horizontal axis in Figure 3 indicates the time and the vertical axis indicates the contact force value obtained by the smart insole. The contact force curve for each detection area includes two parts, as shown in the legend.

The method in this paper focuses on helping people with hand disabilities to perform the actions of daily life, which are a continuous process and are usually performed while the body is at rest, such as drinking water, or while the body is walking, such as wearing a hat while walking. Figure 3a shows the contact force curve of the heel area during walking. During walking, the person repeats the steps of lift-lower-lift in the heel area, and the contact force curve of each cycle is shown in the solid line part. When a person walks, it is difficult to change the contact force state of the heel. The forefoot in contact with the ground must also repeat the lift-lower-lift action, with each cycle accompanied by the creation of a wave peak, as shown in the dotted section. Comparing the wave peaks of the solid and dashed lines, it can be seen that they have very similar characteristics and almost the same amplitude. Figure 3b shows the contact force curve in the forefoot area, whose signal characteristics are similar to those in the heel area. Significantly changing the contact force of these two parts is not only difficult, but also affects the person’s standing balance, so it is not suitable for controlling the assistive hand.

Figure 3c,d show the contact force curves of the hallux and the little toe areas during walking, respectively. The solid line part of them is the contact force value during walking. When the foot is on the ground, the toe can contact the smart insole and generate contact force. Compared with the forefoot and the heel, the toe can easily press the insole at any time when walking or standing, and the movement is also very natural and flexible, easy to perform, and does not affect the balance of the human body. The dotted line is the curve generated by the toe pressing the insole during walking, and wave peaks are formed by pressing the insole. Comparing the curve of the dotted line and the solid line, it can be seen that there is a clear difference in the amplitude of the contact force generated during walking and pressing when walking.

**Figure 4:**
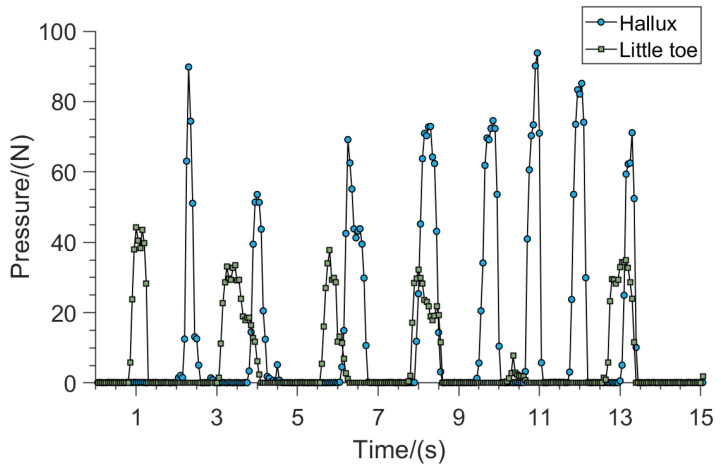
Contact force curve of smart insole during standing.

Ten volunteers were invited to press two areas respectively while their bodies were at rest. It was found that these 10 sets of contact force data obtained showed similar characteristics, so one of them was selected to analyze its characteristics. Figure 4 shows the contact force curve of the hallux and little toe areas when the insole was pressed while standing. As shown in Figure 4, there was a significant peak in the force value when the insole was pressed, while the force amplitude was very small when it was not pressed.

By comparing and analyzing the pressure profiles generated by pressing the smart insoles in different areas of the foot, we find that only the hallux and little toe can extract effective features to distinguish the presence of the pressing action, while other areas have the same features and are difficult to distinguish while not pressing and while pressing. The above results show that whether the human body is walking or standing, the contact force signal of the hallux and the little toe can clearly distinguish whether the insole is consciously pressed, and the contact force signal is less disturbed in the standing or sitting state. Therefore, it can be used to express the person’s intention to control the assistive hand. In contrast, the hallux has higher flexibility than the little toe, and the force signal amplitude generated by pressing the insole is higher, which has higher repeatability and discriminability. Therefore, in this paper, the hallux contact force signal of the foot is selected to control the assisting hand.

## 4. Contact Force Signal Recognition Method of the Foot’s Hallux

### 4.1. Preprocessing of the Contact Force Signal

**Figure 5:**
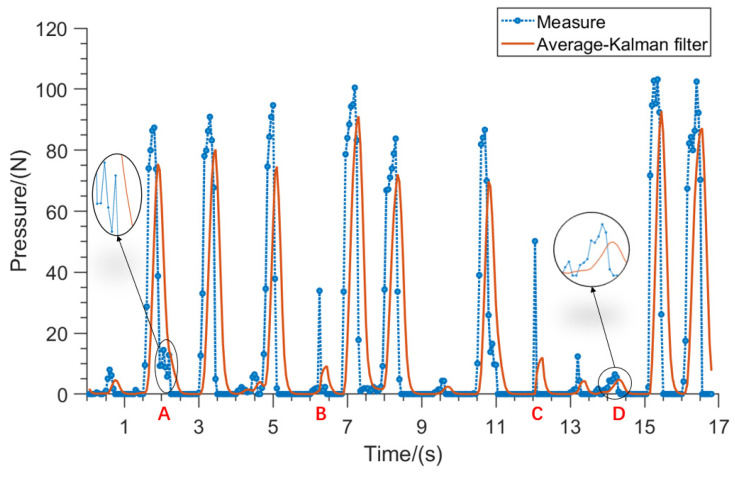
Comparison graph of contact force signal filtering. A to D are the four feature points selected.

The internal circuit noise of sensors and factors such as toe jitter can result in the obtained force signal sequence being contaminated with noise interference. The original contact force data collected by the smart insole are shown in Figure 5 with the blue point curve, and the smoothness is poor. In this paper, a signal processing algorithm combining recursive averaging and Kalman filter is designed, and the filtering process mainly includes two parts as follows.

(1) Recursive average filtering is applied to the original contact force signal, and the processing formula is as
  (1) $$x_{k}=\frac{1}{N}\overset{N-1}{\underset{i=1}{\sum }}y_{k-i}$$
  where $y_{k-i}$ is the measured value of contact force at time *k* − *i*, $x_{k}$ is the recursive average filtering value at time *k*, *N* is the length of the selected queue, and the value range of N is 40–60.
(2) Kalman filtering is performed on the obtained recursive average filtering signal, and the state equation and measurement equation are as
  (2) $$\begin{array}{c} \{\begin{array}{c} x_{k}^{-}=Fx_{k-1}+Bu_{k-1}+w_{k-1}\\ z_{k}=Hx_{k}+v_{k} \end{array} \end{array}$$
  where $x_{k-1}$ is the predicted value of contact force at time *k* − 1, $x_{k}^{-}$ is the estimated value of contact force at time *k*, $u_{k-1}$ is the system input at time *k* − 1, $w_{k-1}$ is the process noise at time *k* − 1, whose covariance is *Q*, and *F* and *B* are system parameters.

In the prediction stage, the optimal contact force estimate at time *k* − 1 is used to predict the contact force value at time *k*, and the error covariance $P_{k}^{-}$ at time *k* is predicted by the previous contact force prediction error covariance $P_{k-1}$ and the process noise *Q*. In the update stage, the optimal estimate of contact force at time *k* is obtained by weighting the predicted value $x_{k}^{-}$ and the observed value $z_{k}-{Hx}_{k}^{-}$ at time *k*.

The original contact force signal data can be obtained as a set of smooth signal sequences after recursive averaging-Kalman filtering, and its curve is shown in Figure 5. The blue dotted line with circular points in the graph represents the original force data curve, while the orange solid line represents the recursively averaged data curve processed by the Kalman fusion filtering algorithm. The comparison between the two curves shows that, after filtering, the noise in the force signal is significantly reduced, as seen from points A and D where the smoothing effect is greatly improved. Additionally, at points B and C, it is evident that the algorithm has a strong suppressive effect on random noise, indicating that this filtering algorithm effectively reduces noise interference. Since the model used in this paper introduces recursive averaging filtering before Kalman filtering, it will involve some delay. This does not affect the experimental results because non-disabled persons or disabled persons let the assistive hand open in advance when grasping objects.

### 4.2. Feature Extraction of the Contact Force Signal

When the hallux is consciously pressing the insole, the tester would detect the wave peak created by the contact force. When the disabled person is in the state of walking, additional wave peaks would be generated to form interference, as shown in Figure 3c. However, the amplitude of the wave peak is obviously smaller compared to the wave peak formed by the pressing action. Due to factors such as weight, there is pressure on the insole when the foot is not pressing on it, and the threshold is for shielding the interference from these factors. In use we only analyze the characteristics of signals greater than the threshold. At the same time, the threshold can also shield the small false contact of the hallux, and it also has an effect on the extraction of the wave peak of the contact curve at rest.

Due to the differences in weight and foot shape of each individual, the specific value of the threshold needs to be derived from each individual’s situation and determined by collecting the maximum value of the contact force detection signal during a period of walking, standing, or sitting. By collecting a segment of force data from the subject prior to the experiment to generate the threshold, this method can be adapted to subjects of different body weights and sizes. When the hallux presses on the insole, the contact force signal will generate a wave peak signal greater than the threshold value, and the peak signal greater than the threshold value can be extracted to determine the conscious pressing action of the hallux. A continuous period of the contact force signal greater than the threshold indicates a pressing action and is recorded as a peak signal. The specific peak extraction algorithm is as follows.

(1) Continuous acquisition of hallux contact force data until the interval between the current data point and the marker point is greater than the scan time *b*. *N* measurements are obtained for filtering, with the marker point being the initial data point of the current cycle or the data point greater than the threshold *t*. The scan time *b* is the shortest acquisition period, adjusted according to the demand for control action time and sensitivity for people with different disabilities.
(2) The times of hallux pressing are judged, the *N* filtered contact force values are recorded as $a_{1},\;a_{2},\;\ldots ,\;a_{N}$, and the specific calculation formula is as
  (3) $$\{\begin{array}{c} g\left(x\right)=\{\begin{array}{c} 1,x<0\\ 0,x\geq 0 \end{array}\\ S=\sum_{i=2}^{N}\frac{g\left(\left(a_{i-1}-t\right)\left(a_{i}-t\right)\right)}{2} \end{array}$$
  where *g*(*x*) is defined as a non-negative function; $a_{i}$ is the *i*-th filtered contact force value, *t* is the contact force threshold, and *S* is the number of wave peaks greater than the threshold in *N* data, a set of data points satisfying $g\left(\left(a_{i-1}-t\right)\left(a_{i}-t\right)\right)=1$, representing a peak signal, reflecting a hallux press action. According to the above algorithm, the number of peak signals that meet the conditions can be obtained, so as to obtain the number of hallux’s pressing actions in the time period, and then control the hand. The above steps can continuously extract the peak features and the number of presses, and realize the continuous control of the hand.

### 4.3. Identifiability Study of Foot Contact Force Signals

The areas of the hallux and the little toe of the foot can both apply recognizable pressure to the smart insole, and the accuracy of identifying pressing actions in each area is used as the preferred basis for haptic control signals. 

Ten volunteers were invited to wear the smart insoles on both feet and stood naturally at rest and pressed the hallux and the little toe areas with both feet to observe the corresponding contact force signal peaks. The recognition results are shown in Table 4, V1 to V10 represent 10 volunteers, whose details are shown in Table 3, L1 and R1 are the left and right foot hallux pressing actions, and L2 and R2 are the left and right foot little toe pressing actions, respectively. Each of the 10 volunteers pressed the smart insole 10 times using the hallux and we counted the number of times the pressing action was correctly recognized. The values in the table indicate the number of obvious wave peaks detected in 10 actions, i.e., the number of successful detections, and the overall accuracy is the average of the successful detection rate of 10 volunteers. From the experimental results of the 10 volunteers, it can be seen that the recognition accuracy of the hallux area signals of the left and right feet are 98% and 99%, respectively, but the recognition accuracy of the signals of the little toe area of the left and right feet are 80% and 78%, respectively, and the different volunteers are more adaptable to the hallux area, so the contact force signals of the hallux area are more suitable as the control signals of this paper with higher recognition accuracy.

## 5. The Fine Control Method of Assistive Hand

### 5.1. Posture Control of the Assistive Hand

**Figure 6:**
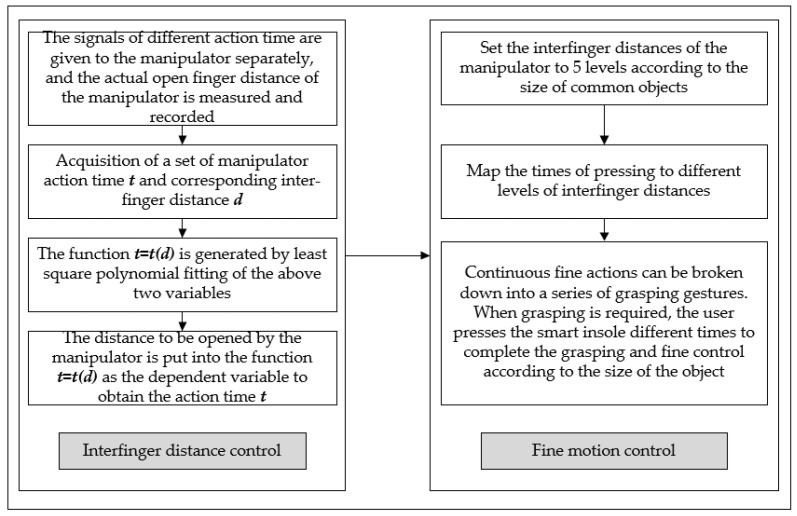
Flow chart of the fine-control method.

The assistive hand posture refers to the relative positional relationship between the assistive hand and the target object while performing a particular operation, and also includes the relationship between the distance between the assisting fingers and the size of the target object. However, the shape and size of the target object vary widely, and assistive hands without the position measurement and control function often lead to task failure. In particular, the probability of task failure is high when the assistive hand performs delicate manipulations on small and flexible objects (such as tying shoelaces and doing up and undoing a zip). The fine-motion control method of the assistive hand is shown in Figure 6, which mainly includes two parts: interfinger distance control and fine-motion control. Firstly, the functional relationship between *t* and *d* is obtained by collecting a set of data of robot action time *t* and interfinger distance *d*. Then, the interfinger distance is divided into several levels according to the common object size, and this paper takes 5 levels as an example, and the number of presses is mapped to different inter-finger distances, so the user can finely control the movements of the assistive hand. Therefore, it is very necessary to control the distance between the fingers of the assistive hand. In this paper, a time-finger-distance fitting algorithm is developed.

First, let the finger of the assistive hand be completely closed, then use the time $t_{i}$ to open the distance to $l_{i}$, and repeat the experiment to collect *N* groups of data ($l_{i}$, $t_{i}$), as shown in Table 5. The time to completely open is $t_{A}$, and when the power supply voltage is constant, $t_{A}$ is close to constant.

The steps of this algorithm are as follows.

**Figure 7:**
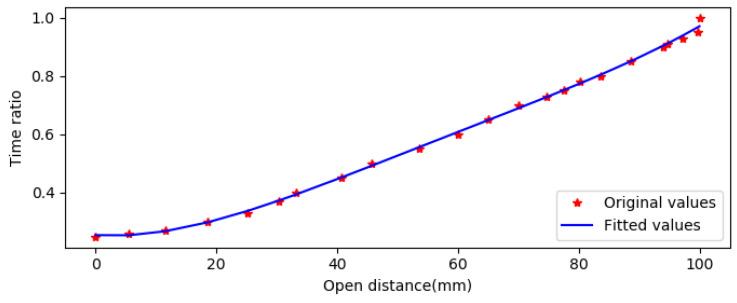
The fitting curve of the interfinger distance to time ratio.

(1) Define the time ratio:
  (4) $$d_{i}=\frac{t_{i}}{t_{A}}$$
(2) Establish the target polynomial of interfinger distance to time ratio:
  (5) $$d=(\Sigma_{i=1}^{M}w_{i}l^{i})+b$$
  where $w_{i}$, b is the fitting coefficient and *M* is the power.
(3) The matrix form of the error function is solved, and the formula is
  (6) $${\left|\left|Lw-d\right|\right|}_{2}$$
  where $w=\{b,w_{1},w_{2},\ldots ,w_{M}{\}}^{T}$ is the coefficient vector, $d=\{d_{1},d_{2},\ldots ,d_{N}{\}}^{T}$ is the annotation vector, ***L*** is the input matrix, and the formula is
  (7) $$L=\left[\begin{array}{ccccc} 1 & l_{1} & l_{1}^{2} & \ldots & l_{1}^{M}\\ 1 & l_{2} & l_{2}^{2} & \ldots & l_{2}^{M}\\ \ldots & \ldots & \ldots & \ldots & \ldots \\ 1 & l_{N} & l_{N}^{2} & \ldots & l_{N}^{M} \end{array}\right]$$
(4) To solve the parameter vector ***w***, the formula is
  (8) $$\frac{\partial {\left|\left|Lw-d\right|\right|}_{2}}{\partial w}=2L^{T}Lw-2L^{T}y=0$$
  where the partial derivative of the error function with respect to the parameter ***w*** is zero. Put $w=\{b,w_{1},w_{2},\ldots ,w_{M}{\}}^{T}$ into Equation (5) to obtain the fitting objective polynomial.
(5) Determinate the power *M*. The value of *M* is less than *N*, and *M* is again increased to determine the optimum value according to SSE and R2. As shown in Table 6, the SSE can reflect the correlation of polynomials, and when *M* is taken as 2 or 3, the SSE is large, the fit is not satisfactory, and there is more room for improvement. When *M* is set to 4, SSE is very small, and when the value of *M* continues to increase, the variance changes are not obvious. Considering the computational complexity, *M* is set to 4, and the fitting curve is shown in Figure 7.
(6) The interfinger distance and the ratio of opening and closing time are expressed in
  (9) $$d=0.01441l^{4}+0.002019l^{3}-0.001985l^{2}+0.2644l+0.5976$$
  where *l* is the set target interfinger distance, and the unit is mm.
(7) Calculate the opening and closing time, and the calculation formula is
  (10) $$t_{open}=d\left(l\right)\times t_{A}$$
  where $t_{open}$ is the opening time required under *l* opening distance.

### 5.2. Design of the Coding Instructions for the Posture Control of the Assistive Hand

According to the method of 4.1, by accurately controlling the action time of the assistive hand, the interfinger distance can be accurately controlled, so that the assistive hand can maintain the corresponding grasping posture. In this paper, the foot hallux contact force is selected as the control signal of the hand action. Considering the difficulty of human memory encoding and the size of common objects, this paper proposes a coding method of five levels of control of interfinger distance. The maximum distance between the fingers of the assistive hand is 100 mm, and the five distance values are graded as 0 mm, 25 mm, 50 mm, 75 mm, and 100 mm. The hallux action mapping method is defined as shown in Table 7. The assistive hand is closed without hallux pressing in the next data cycle if the current state is open, and the number of hallux presses in the current state corresponds to different distances. At the same time, the error of the control experiment is given. According to the results of Table 4, it can be concluded that the proposed control algorithm has good control accuracy and good performance for different interfinger distances, which can help the assistive hand to complete the adaptive grasping of objects of different sizes and shapes.

## 6. Experiment

### 6.1. Continuous Fine Motor Test for Disabled Persons

This method is designed to help people with upper extremity disabilities to re-establish hand function, mainly those who only have upper extremity disabilities but are otherwise non-disabled. Users are indeed interested in many movements. However, due to the limitation of space and the possibility of showing the completion of movements when the lower limbs are in different states, we carefully designed the experimental movements in the paper. We contacted the local disability federation to help recruit people with only hand disabilities. In this paper, five volunteers with impairments of one hand but otherwise non-disabled and five volunteers with impairments of both hands but otherwise non-disabled were invited for confirmatory experiments. All of them have only upper limb disabilities. The learning cost was low for all subjects. Volunteers learnt to use the system very easily and it consisted of two main parts. First, they wore smart insoles and prosthetic hands to adapt to sitting, standing, and walking states; then, they learnt to complete halluces to press insoles in the above three states. They learned in less than an hour and were able to use it normally. According to Table 7, volunteers can control the opening or closing of the assistive hand by pressing the insole with their hallux. When they wanted to activate the assistive hand, they only needed to press the insole. After pressing, the assistive hand will open to the corresponding interfinger distance. Each volunteer used the disabled hand in the same way. They all wore the smart insole inside the shoe and the artificial hand on the upper limb, and then pressed the smart insole to control the assistive hand. As can be seen from Table 8, all subjects showed similar experimental results, and due to the limitation of space, the experimental procedure of volunteer with impairments of one hand and both hands were randomly selected to focus on the analysis. The selected volunteer with impairments of one hand is a young woman with a missing right wrist and usually wears a cosmetic hand. She can perform common daily activities with her left hand, but it is difficult to perform them with one hand in some situations (such as tying shoes and dressing clothes). The selected disabled volunteer with impairments of both hands is a young male who has lost 90% of both forearms due to electrocution as a child, making it difficult for him to complete daily living activities, many of which require the cooperation of his mouth. The volunteer with impairments of one hand has been disabled for 10 years, is 38 years old, and weighs 59 kg. The subject with both-handed disability has been disabled since childhood, is 31 years old, and weighs 68 kg. 

The two volunteers performed daily behavioral experiments, including carrying objects, grasping small objects, drinking water, wearing hats, wearing coats, undoing a zip, tying shoelaces, wearing masks, and putting things into pockets. These actions could cover most of the activities in daily life or are similar to them. These actions were divided into one-time grasping actions (such as drinking water) and continuous grasping actions (such as wearing coats, tying shoelaces, taking a mask out of a pocket, and putting it on) according to whether they grasped the object multiple times.

Taking drinking water, tying shoelaces, and taking off coats as examples to show the experimental results, the first set of actions is a one-time grasping action, and the latter two sets of actions are very common in daily life but very complex continuous actions. First, the experimental process and the corresponding hallux pressing behavior were introduced through the contact force curve of the smart insole, and then the quality of action completion was analyzed to show the control effect of the assistive hand. This paper had performed a large number of experiments, which could include most of the daily actions, and also included the grasping of different sizes and shapes of objects. Due to the limitation of space, only three representative actions were selected to perform a focused analysis.

#### 6.1.1. Drinking Water Experiment

**Figure 8:**
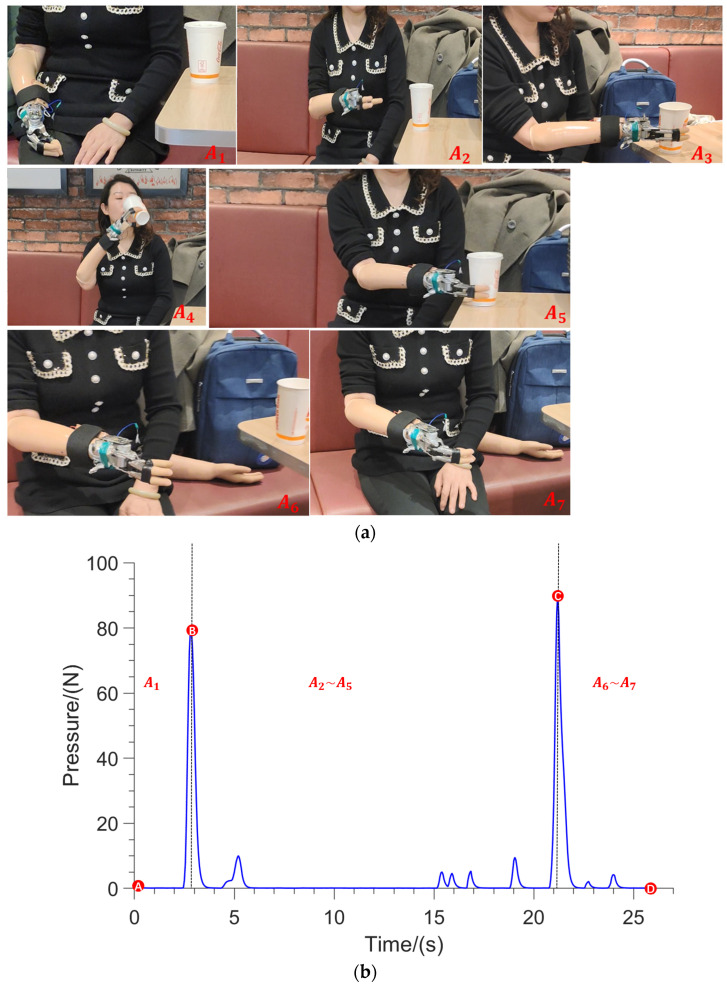
The experiment of drinking water for the disabled people. (**a**) Experimental process photos, A_1_ to A_7_ are different states of the experiment; (**b**) contact force curve of experimental data, A to D are the characteristic points.

Drinking water is a very frequent action, which represents many types of one-time grasping actions. After wearing the right assistive hand, the female volunteers performed the action of drinking water in the sitting position. The experimental procedure was recorded as shown in Figure 8a, and the contact force data of the experimental procedure are shown in Figure 8b.

The drinking experiment can be broken down into actions A_1_ to A_7_ in Figure 8a, where A_1_ is the initial preparation stage. The hallux pressed the insole at A_2_ and A_3_, generating a wave peak at point B in the contact force graph in Figure 8b, and the assistive hand opened and moved toward the cup to grasp it. The artificial hand moved the cup toward the mouth to finish drinking at A_4_. The cup was placed on the table at A_5_. The hallux pressed again at A_6_ and A_7_, generating a wave peak at point C in Figure 8b, and the assistive hand opened and released the water cup to return to the initial state. The whole process included drinking and placing the cup, there was a one-time grasping action and two opening actions of the assistive hand, and the disabled person pressed the smart insole twice.

#### 6.1.2. Tying Shoelaces Experiment of Volunteer with Impairments of One Hand

**Figure 9:**
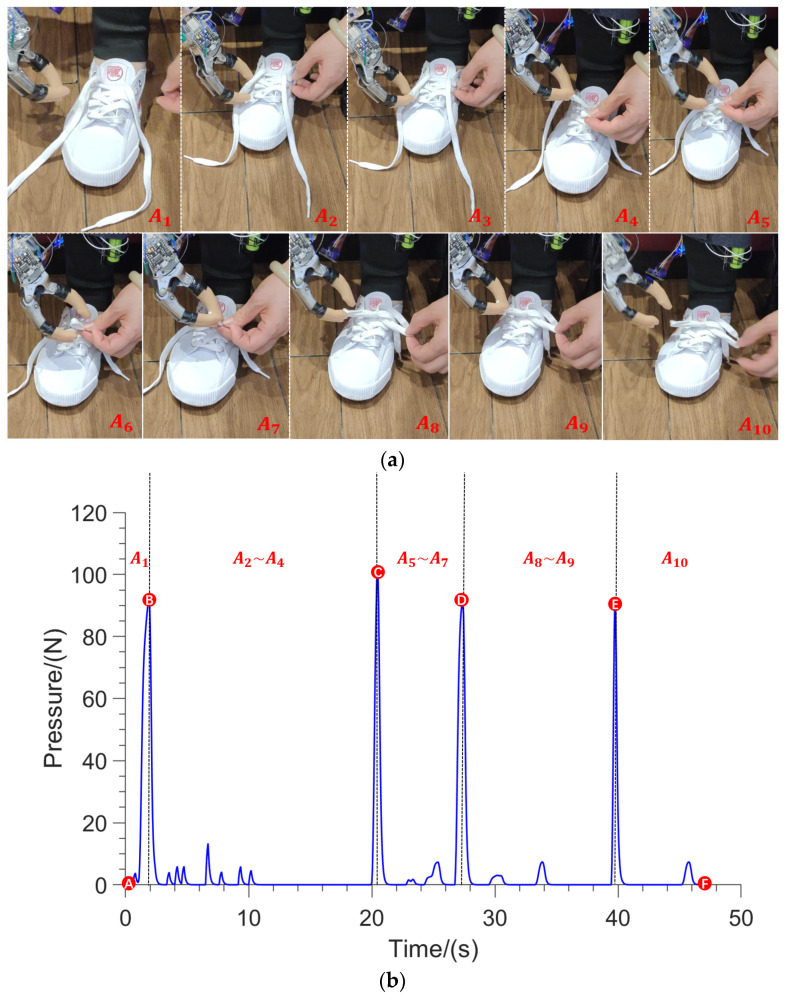
The experiment of volunteers with impairments of one hand tying shoelaces. (**a**) Experimental process photos, A_1_ to A_10_ are different states of the experiment; (**b**) contact force curve of experimental data, A to F are the characteristic points.

Figure 9a shows the action process of tying shoelaces with the cooperation of the left and right hand. Figure 9b shows the data of the recording process.

Figure 9a shows the experimental process of this action, and Figure 9b shows the contact force curve generated by the subject in a shoelace-tying cycle. Different moments in the curve correspond to the experimental process, and the curve represents the value of the hallux contact force. Due to the long time of the whole action, Figure 9 and the following contact force data graph mainly show the contact force data near the steps in the experimental process photos. The whole cycle can be divided into five parts separated by the black line. The numbered image above each part represents the current action state of the disabled person. Point A is the initial state where the hallux was held still and the hands were close to the shoelace until state B. At points B, C, D, and E, the hallux was suddenly pressed and then released, and the contact force suddenly increased. At this time, the assisting hand opened and moved toward the shoelace and performed the action with the left hand. Except for the pressing state of the hallux near points B, C, D, and E, the hallux was in a static state at other times. There was an obvious pressing behavior at the transformation points in the above action phases, and the signal curve showed an obvious peak state.

#### 6.1.3. The Experiment of Taking off the Coat for the Volunteer with Impairments of Both Hands

**Figure 10:**
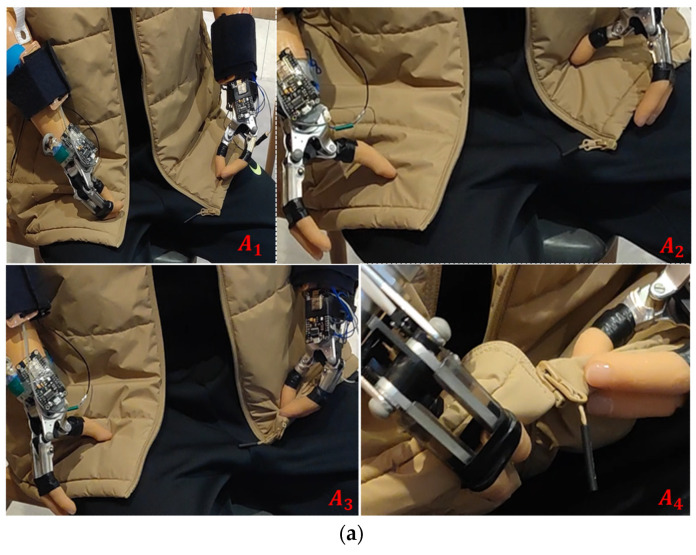

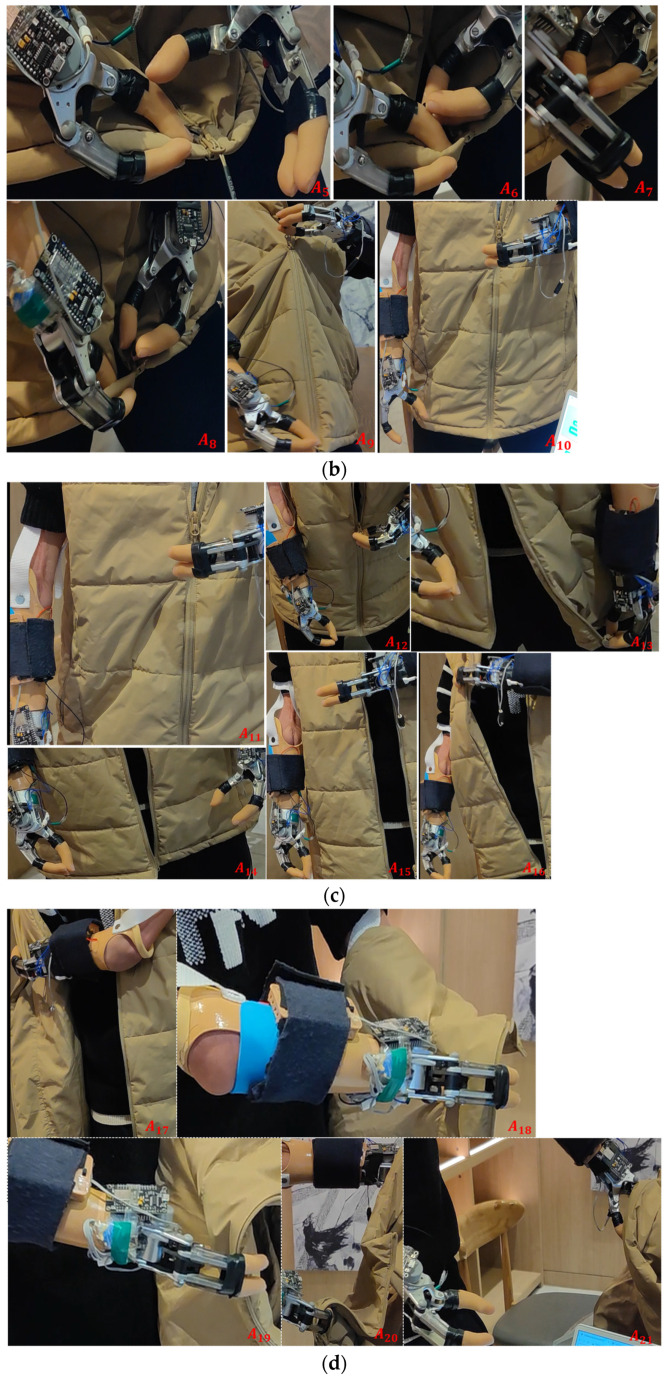

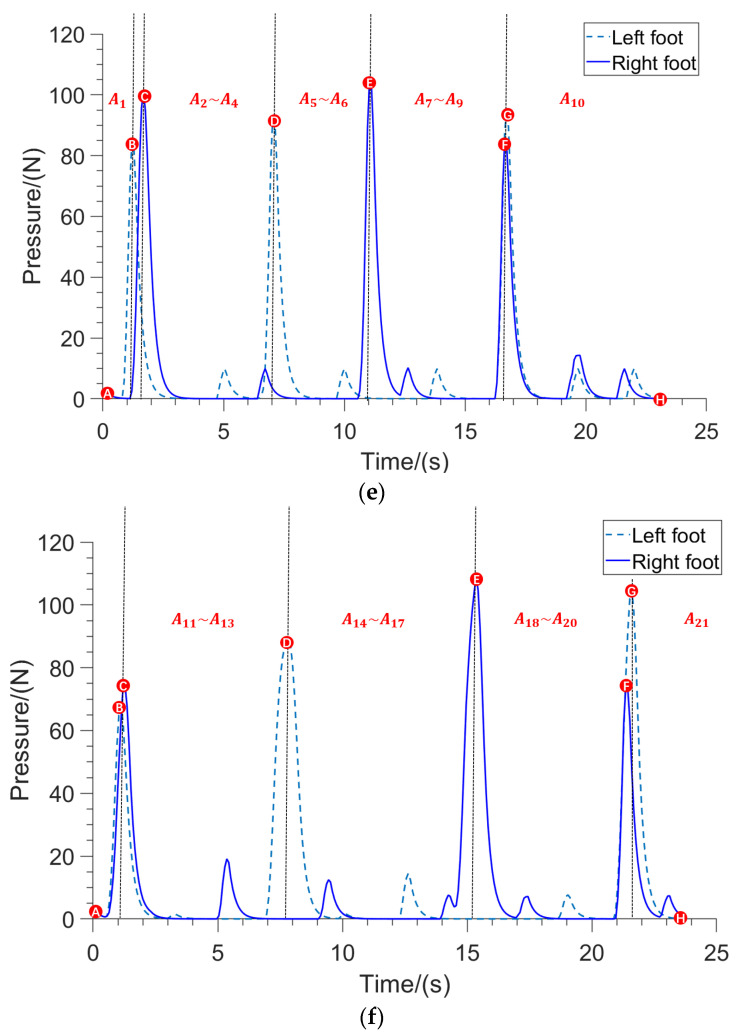
The experiment for the volunteer with impairments of both hands. A_1_ to A_21_ are different states of the experiment. (**a**) Experimental process photos in preparing; (**b**) experimental process photos in doing up a zip; (**c**) experimental process photos in undoing a zip; (**d**) experimental process photos in taking off; (**e**) contact force curve of zipping; and (**f**) contact force curve of taking off the coat, A to H are the characteristic points.

The male volunteer is a volunteer with impairments of both hands. The assistive hand was worn on his upper arm before the experiment, and then he performed the action of taking off the coat, and the process data were recorded and analyzed. The action was more complicated, so it was divided into two parts for analysis, as shown in Figure 10e for the zipper contact force curve, and Figure 10f for the contact curve of taking off the coat. Figure 10a–d show the experimental procedure photos corresponding to different moments of the curves.

Figure 10b shows the data of the disabled person’s zipper-pulling process: the light blue dashed line is the contact force signal curve of the left foot, the dark blue solid line is the contact force signal curve of the right foot, and the whole action cycle is divided into five parts, namely AB, BD, DE, EG, GH. Except for the that the left hallux pressed at the three points B, D, and G, and the right hallux pressed at the three points C, E, and F, the rest of the time both halluces were at rest. The AB section was the initial stage: the hands moved to the bottom of the clothing zipper. In the BD section, the left hand grasped the left side of the zipper. In the CD section, the right hand grasped the right side of the zipper clothes, and the zipper was aligned. In the DG section, the left hand grasped the zipper ring. In the EF section, the right hand adjusted the posture, and the zipper was pulled. At FH and GH, the hands were released and the zipper action was completed. There was a clear pressing behavior at the transition points of each action phase mentioned above, and the signal curve shows a clear peak state.

Figure 10c shows the data of the process of undressing for the disabled person. The light blue dashed line is the contact force signal curve of the left foot, the dark blue solid line is the contact force curve of the right foot, and the whole action cycle is divided into five sections by the black line, which are AC, CD, DE, EF, and FH. Except for the fact that the left hallux pressed at three points C, D, and G, and the right hallux pressed at three points B, E, and F, the rest of the time both halluces were at rest. AC section was the initial stage, the left hand went to the zipper and the right hand went to the bottom of the clothes. In the CD section, the left hand grasped the zipper ring. In the BE section, the right hand grasped the bottom of the garment and the left hand pulled the zipper down until both sides of the garment were unzipped. In the DG section, the left hand grasped the right side of the garment. In the EF section, the right hand was released and the left hand removed the garment. At the FH and GH sections, both hands were released and the garment was placed on the table. At the transformation points of each of the above action stages, there was obvious pressing behavior, and the signal curves all showed obvious wave states.

## 7. Results and Discussion

The criteria used to evaluate the intention recognition and control performance of the assistive hand are the accurate opening of the assistive hand to the corresponding interfinger distance in each state of the action and the quality of the completed action. As shown in the experimental procedure in Section 6, a desired task can be broken down into multiple grasping actions, so the desired task can be achieved by completing all grasping tasks. The grasping action involves the opening of the assistive hand, so the accuracy of the correct opening of the assistive hand in the whole task, i.e., the ratio of the number of correct opening times to the number of required opening times, can reflect the accuracy of the desired task. Table 8 shows the results of the experiments in which volunteers with impairments of one hand and both hands completed the actions separately, and both completed the nine movements in the table. The open times refer to the number of openings of the assistive hand needed to complete the action correctly. The total accuracy rate is the overall accuracy rate of five volunteers completing the task, i.e., the ratio of the average number of correct opening times of the five volunteers and the number of correct opening times needed. V1 to V5 represent five volunteers, respectively, and their corresponding values indicate the average number of correct openings when volunteers repeatedly completed the corresponding actions five times. From the experimental results, it can be seen that the accuracy rate of completing many kinds of actions for volunteers with impairments of one hand is 100%, and the average accuracy rate of the five kinds of actions is 99.20%. Volunteers with impairments of both hands can achieve 100% accuracy rate when completing actions such as putting on a mask and putting on and taking off a hat, and the total accuracy rate of the four kinds of actions reaches 98%. There are two situations that can occur when the disabled hand fails to accurately recognize the user’s intention: the hand may fail to open in time or fail to close in time. In either case, the disabled person only needs to press their halluces again to continue the action.

From the above results, it can be seen that both volunteers with impairments of one hand and both hands can perform the common continuous fine motor actions easily with the assistive hand. Each action was repeated five times by five volunteers, totaling 25 times. The accuracy rate of all nine actions was high, and there were few errors even when performing actions such as carrying objects, so the assistive hand has high stability and ease of use. The actions selected in the experiment include one grasping and continuous grasping movements, covering common movements such as grasping, wearing, moving, and fine grasping, with high accuracy rates, indicating that the method has high universality. The smart insole can be placed inside the shoe, and the WIFI module is small enough to be attached to the insole and the robot, so it is unobtrusive.

The control system designed in this paper was applied to different actions of volunteer with impairments of one hand and both hands. Each experiment could be completed with high quality, and the accuracy of action completion was 99.20% and 98%, respectively. This shows that the system has good performance and universality and can efficiently complete daily actions.

## 8. Conclusions

This paper proposes an assistive hand control method based on the contact force signal of the hallux, which encodes the contact force signal and the smart insole to express the human grasping intention for various objects and can effectively control the assistive hand to realize the grasping of various objects. It can help volunteers with both impairments of one hand and both hands to perform a variety of continuous fine actions, such as moving objects, grasping small objects, drinking water, putting on and taking off hats, putting on and taking off coats, zipping, tying shoelaces, wearing masks, taking out and putting into pockets, and so on. The experimental results show that this method is feasible and universal. Compared with bioelectrical signals such as EMG signals and EEG signals to control the assistive hand, this method has advantages in terms of ease of use, anti-interference, and unobtrusiveness, but requires the involvement of the foot. Compared with voice signals and motion signals to control the assistive hand, this method has advantages in terms of stability and multiple scenarios. However, this method is only used for people with hand disabilities only. If a person with hand disabilities also has disabilities in other body parts, such as feet, the function of this system will be limited and ineffective. The threshold values in this method are set somewhat conservatively, and are determined based on the maximum value of a set of pressure data of the volunteer without pressing the insole, and this method sometimes needs to be adjusted based on experience. A more suitable threshold can be set by building a set of models based on parameters such as the user’s weight, height, and foot shape. Limited by the difficulty of finding suitable volunteers, this method invited 10 volunteers to participate in the experiment, and if more volunteers were found to participate in the experiment, this method would have higher generalizability. These are the next steps to be done.

The assistive hand control is a typical human-in-the-loop control method. Through contact force coding, the disabled person can directly express the control intention of the assistive hand and play a perceptual and decision-making role in the loop. Since the human is the dominant player in the loop, the method has higher stability compared with the control method based on EMG and EEG signals. The proposed method avoids the complex modeling of “human” and also avoids the high tension of human muscles and attention and skin discomfort. Compared with speech signals, the method in this paper is more covert, which not only avoids the embarrassment of “talking to oneself” in social situations but also is less susceptible to interference from the environment. In addition, the method in this paper is less costly and has better scalability.

In order to further improve the ease of use and unobtrusiveness of this system, we consider integrating the smart insole into the shoe and using a lighter weight prosthetic hand, which will make the system more accessible to more disabled people and reduce the learning costs for commercialization. In the future, we will continue to improve the intelligence level of the assistive hand so that it can automatically recognize the type of target object. This will enable the assistive hand to automatically adapt to different operational targets, thus simplifying its use.

## Figures and Tables

**Table 1:** Summary table of background.

Study	Data Sources	Applications
Feleke et al. (2021) [2]	EMG	Hand trajectory prediction
Zheng et al. (2022) [3]	EMG	Identify and predict hand movements
Nazari et al. (2023) [4]	EMG	Assistive hand control
Jiang et al. (2022) [7]	EMG Inertial measurement unit (IMU)	Hand gesture recognition
Zhang et al. (2022) [8]	EMG	Hand gesture recognition
Yang et al. (2021) [9]	EMG	Hand gesture recognition
Triwiyanto et al. (2022) [10]	EMG	Assistive hand control
Wang et al. (2022) [11]	EEG	Hand movement recognition
Fu et al. (2023) [12]	EEG	Hand movement recognition
Ofner et al. (2019) [13]	EEG	Hand gesture recognition
Tang et al. (2023) [14]	Smart insole	Health analysis
Kromołowska et al. (2023) [15]	Smart insole	Sport monitoring
Vijayaragavan et al. (2019) [16]	Voice	Assistive hand control
Cui et al. (2022) [17,18]	IMU	Assistive hand control
Li et al. (2022) [19]	EMG, IMU	Human movement classification
Chakraborty et al. (2021) [20]	Vision	Human movement recognition
Rasel et al. (2019) [21]	Vision	Hand gesture recognition
Sarcar et al. (2021) [22]	Vision	Upper limb movement classification

**Table 2:** Control logic of artificial hand.

Input Signals	High Level Signal to Line B	Low Level Signal to Line B
**High level signal to line A**	-	Open
**Low level signal to line A**	Close	Stationary

**Table 3:** Details of the 10 volunteers.

	V1	V2	V3	V4	V5	V6	V7	V8	V9	V10
Level of disabilities	L1 ^1^	L2 ^2^	L3 ^3^	L2	L1	L3	L2	L3	L1	L1
Age	23	22	23	38	23	24	25	24	31	23
Gender	male	male	male	male	male	female	female	female	female	female

^1^ L1: Volunteers with impairments of one hand but otherwise non-disabled. ^2^ L2: Volunteers with impairments of both hands but otherwise non-disabled. ^3^ L3: Volunteers with non-disabled.

**Table 4:** Recognition results of different foot areas.

	V1	V2	V3	V4	V5	V6	V7	V8	V9	V10	Total Accuracy
L1	10	10	9	10	10	10	10	10	10	9	98%
L2	8	7	8	9	8	8	8	9	8	7	80%
R1	10	10	10	10	9	10	10	10	10	10	99%
R2	7	8	7	8	9	8	8	7	9	7	78%

**Table 5:** Opening time data of assistive hand.

Size (mm)	Time Ratio	Size (mm)	Time Ratio	Size (mm)	Time Ratio	Size (mm)	Time Ratio
100	1	83.62	0.8	60	0.6	25.14	0.33
99.8	0.95	80.19	0.78	53.68	0.55	18.59	0.3
97.31	0.93	77.49	0.75	45.8	0.5	11.54	0.27
94.74	0.91	74.63	0.73	40.74	0.45	5.46	0.26
94.01	0.9	70	0.7	33.14	0.4	0	0.25
88.71	0.85	65	0.65	30.36	0.37		

**Table 6:** Error table of the *M* value.

M Value	1	2	3	4	5	6
SSE	0.0207	0.0041	0.0029	0.0018	0.0017	0.0017
R^2^	0.9852	0.997	0.9979	0.9987	0.9988	0.9988

**Table 7:** Mapping method and control error of the hallux pressing action.

The Current State of the Assistive Hand	Hallux Press Action (In One Data Cycle)	Next State of the Assistive Hand	Mean of Actual Distance (mm)	Absolute Error (mm)	Relative Error
Open	Press 0 time	Closure	0	0	0%
Closure	Press 1 time	Open to a = 25 mm	28	3	12%
	Press 2 times in a row	Open to b = 50 mm	52	2	4%
	Press 3 times in a row	Open to c = 75 mm	70	5	6.67%
	Press 4 times or more in a row	Open to d = 100 mm	95	5	5%
	Mean			3	5.53%

**Table 8:** Experimental results of various actions.

	Movements	Open Times	V1	V2	V3	V4	V5	Total Accuracy
Volunteers with impairments of one hand	Grasp small objects	6.0	6.0	6.0	5.8	6.0	6.0	99.33%
	Carrying objects	5.0	5.0	5.0	5.0	5.0	5.0	100.00%
	Drink water	7.0	7.0	6.8	7.0	7.0	7.0	99.43%
	Doing up and undoing a zip	6.0	6.0	6.0	6.0	6.0	6.0	100.00%
	Tying shoelaces	6.0	6.0	6.0	5.2	6.0	6.0	97.33%
	Mean							99.20%
Volunteers with impairments of both hands	Wear a mask	5.0	5.0	5.0	5.0	5.0	5.0	100.00%
	Put things in your pockets	10.0	9.2	10.0	10.0	10.0	8.4	95.20%
	Wearing and removing a hat	8.0	8.0	8.0	8.0	8.0	8.0	100.00%
	Wear and take off your coats	9.0	9.0	9.0	9.0	8.2	9.0	98.22%
	Mean							98.00%

## Data Availability

The data presented in this study are available on request from the corresponding author.
